# Orchids from Rio Grande Do Sul, Southern Brazil: What We Know and What We Still Have to Know to Ensure Their Conservation

**DOI:** 10.3390/plants15203122

**Published:** 2026-10-12

**Authors:** Rodrigo B. Singer, Nelson Barbosa Machado Neto, Wendy Carolay Navarro Romo, Amanda Magalhães de Sena, Ceci Castilho Custódio, Rosana Farias-Singer

**Affiliations:** 1 Botany Department and Graduate Program in Botany, Universidade Federal do Rio Grande do Sul (UFRGS), Av. Bento Gonçalves 9500, Block IV, Porto Alegre 90035-003, Brazil; 2 Biology College, Universidade do Oeste Paulista (UNOESTE), Campus II, Rodovia Raposo Tavares, km 572, Limoeiro–Bloco B2, Presidente Prudente 19067175, Brazil; nbmneto@unoeste.br (N.B.M.N.); amanda.magalhaes755@gmail.com (A.M.d.S.); ceci@unoeste.br (C.C.C.); 3 Graduate Program in Botany, Universidade Federal do Rio Grande do Sul (UFRGS), Av. Bento Gonçalves 9500, Block IV, Porto Alegre 90035-003, Brazil; orchidsurpilay@gmail.com; 4 Porto Alegre Botanical Garden, SEMAI/RS, Porto Alegre 90119-900, Brazil; rosana-singer@sema.rs.gov.br

**Keywords:** Orchidaceae, Atlantic Rain Forest, Pampa, *Cattleya*, *Gomesa*, Laeliinae, Oncidiinae, Chloraeinae, Spiranthinae, germplasm banks

## Abstract

Rio Grande do Sul (Brazil) has significant orchid biodiversity and is the birthplace of several cultivated Epidendroideae species, especially in the genera *Cattleya* (Laeliinae) and *Gomesa* (Oncidiinae), mostly from the threatened Atlantic Rainforest. In contrast, the grassy Pampa Biome has remarkable diversity of terrestrial Orchidoideae species, especially in the subtribes Spiranthinae, Chloraeinae (*Chloraea* and *Bipinnula*), and Orchidiinae (*Habenaria*); many have cultivation potential. Considering the published literature with information relevant to the in situ and ex situ conservation of these species, the following patterns arose: (1) the basic reproductive biology (natural pollination, breeding systems) is better known in Epidendroideae orchids, (2) reliable protocols to store and germinate seeds are available for *Cattleya* species, but are still being developed or have to be developed for other orchid groups, (3) so far, reintroduction experiments have only been performed for *Cattleya intermedia*, a commercially relevant species, and (4) several endangered species are not yet represented in conservation units. In addition, we present new information pertinent to seed germination in some taxa. In this context, we thus strongly suggest the following steps: (1) to perform considerable additional fieldwork to locate still overlooked populations, especially for threatened species, (2) the creation of Conservation Units, especially for endangered species not protected yet in situ, (3) promoting ex situ protection by cultivation in Botanical Gardens or analogue collections, coupled with the storage of seeds and/or pollinia in germplasm banks, and (4) encouraging local researchers to propagate native species and perform reintroduction/restoration experiments.

## 1. Introduction

Rio Grande do Sul is Brazil’s southernmost State and holds ca. 346 orchid species across 90 genera [1], collectively representing ca. 13% of Brazil’s species and ca. 36.58% of its genera [1]. This Brazilian State is built up by two Biomes: (1) the mostly tropical Atlantic Rainforest (aka ‘Mata Atlântica’) and (2) the grassy, mostly temperate Pampa Biome. These biomes contrast sharply in biodiversity: the Mata Atlântica is especially rich in epiphytes. In contrast, the Pampa is remarkable for terrestrial orchids growing among grasses or around rocky outcrops, with comparatively few forested areas and fewer epiphytes [2]. From the total of the recorded 346 orchid species, 322 of them dwell within the Atlantic Rain Forest Biome and 76 within the Pampa Biome, with 52 species being shared by both Biomes [1]. Notably, the Pampa Biome is well represented in neighbouring Argentina and Uruguay, but Rio Grande do Sul is the only Brazilian State with this vegetation assemblage. Notably, both the Pampa and the Mata Atlântica in Rio Grande do Sul host plant species with clear Andean affinities [3,4], most evident in the ‘Floresta Ombrofila Mista’, a Mata Atlântica subtype dominated by stands of *Araucaria angustifolia* (Bertol.) Steud. (Araucariaceae) and other floristic elements adapted to wet, cool conditions, such as *Fuchsia regia* (Onagraceae) and *Gunnera manicata* Linden ex Delchev. (Gunneraceae) which mirror Andean counterparts [3].

Orchids are known for their often complex, specific habitat and pollination requirements, and rank among the most threatened plant groups due to habitat loss and illegal trade. Understanding orchid breeding systems and natural pollinators is an important prerequisite for in situ conservation [5]. Depending on their ability (or lack thereof) to produce fruit and viable seed without pollinators, orchids can be divided into pollinator-dependent and pollinator-independent groups. Pollinator-independent orchids are most common in conditions where pollinators are rare (e.g., high altitudes, cold climates, etc.). However, some pollinator-independent orchids also reproduce sexually; in these cases, pollinator independence is a facultative way to produce fruit in the absence of pollinators and does not represent a ‘dead end’. Pollinator-dependent orchids, by contrast, cannot set fruit or produce viable seed without pollination mediated by animal pollen vectors [5]. Pollinator-dependent orchids can be divided into two main categories: self-compatible species (able to set fruit and viable seed following self-pollination) [6,7,8,9] and self-incompatible species (unable to set fruit and viable seed following self-pollination) [5]. However, even in self-compatible species, seeds from cross-pollinations often perform better in terms of viability (presence or absence of an embryo) and development [7]. Very few orchids native to Rio Grande do Sul have been evaluated globally according to IUCN guidelines. However, the SEMAI/RS (Secretaria Estadual do Meio Ambiente e Infraestrutura do Rio Grande do Sul) produced a local evaluation of the Flora in agreement with IUCN standards [10], identifying 25 threatened species for the State. To date, this is the most up-to-date source for this orchid flora. The list was supposed to be updated regularly, but it has not been updated yet. This contribution addresses the recent literature on orchids native to Rio Grande do Sul whose pollination, breeding system, and propagation have been studied. In addition, we address issues relevant to their ex-situ conservation: the presence/absence of these orchids within Conservation Units and Botanical Gardens; the existence (or lack) of protocols for large-scale propagation; and the existence of reintroduction trials/protocols. The questions behind this contribution are: (1) What do we know about the pollination and breeding systems of Orchidaceae native to Rio Grande do Sul? (2) Are these orchids endangered? (3) Are these orchids protected within Conservation Units? (4) Are there reliable protocols for the storage and propagation of these orchids by seed? (5) Have these orchids been successfully reintroduced in Nature by appropriate protocols?

## 2. Results

For the sake of practicality, results are grouped by orchid group (subtribes). However, information on seed measurements, germination, and conservation is presented collectively (for all orchid groups) in Section 2.7. All orchid species studied so far in Rio Grande do Sul are pollinator-dependent [4,6,7,8,9,11] and rely on different animals for natural pollination, with orchid groups that are essentially self-compatible [4,6,7,8,9] or self-incompatible [5,11].

### 2.1. Laeliinae Orchids (Cattleya spp. and Relatives)

Rio Grande do Sul is home to several *Cattleya* (Figure 1) species and varieties already appraised in horticulture. All these species dwell within the Atlantic Rain Forest domain, with *Cattleya intermedia* Graham, *Cattleya purpurata* (Lindl. & Paxton) Van den Berg, and *Cattleya tigrina* A. Rich. ex Beer growing at lower altitudes (up to 400 m). Conversely, *Cattleya coccinea* Lindl. grows at higher altitudes (900 m or more), mostly within the ‘Floresta Ombrofila Mista’ (forests dominated by *Araucaria angustifolia*) [7]. All four of these taxa are widely cultivated, either as species or as selected forms. *Cattleya coccinea*, in turn, is often used in hybridisation as a source of red/orangish tones. The sister species *Cattleya cernua*, also present in the RS flora [1,12], is heavily collected because of its multiflorous habit and the dwarf size it confers on offspring [13]. Two native *Epidendrum* L. species (*Epidendrum densiflorum* Hook. and *Epidendrum fulgens* Brogn. have been studied for their pollination biology and breeding systems [8,11].

#### 2.1.1. Current Protection and Threats

For the *Cattleya* species mentioned above, the SEMAI/RS has proposed the categories summarised in Table 1. No one has evaluated these species globally. Remarkably, researchers at Feevale University have successfully reintroduced *Cattleya intermedia* localities of Rio Grande do Sul [13,14,15].

#### 2.1.2. Breeding Systems and Natural Pollination

Table 2 summarises the known pollinator groups and breeding systems of the *Cattleya* and *Epidendrum* studied in Rio Grande do Sul. Whereas all studied species are pollinator-dependent, not all are self-compatible; *Epidendrum densiflorum* being self-incompatible, instead. Nevertheless, in *C. coccinea* at least, the proportion of embryo-bearing seeds is higher in fruits produced through cross-pollination (Table 2). Overall, natural fruiting success is low (≤6.7%) [7].

### 2.2. Oncidiinae Orchids (Gomesa spp. and Relatives)

In its current delimitation, *Gomesa* R. Br. is a monophyletic yet heterogeneous orchid genus that includes most Brazilian species formerly placed in *Oncidium* Sw., as well as the genera *Baptistonia* Barb. Rodr., *Coppensia* Dumort., *Ornithophora* Barb. Rodr., *Rodrigueziella* Kuntze, *Rodrigueziopsis* Schltr. and *Theodorea* Barb. Rodr. [5,16]. The only consistent diagnostic feature is the fusion of the lateral sepals (a character that also occurs outside the genus, for instance in sympatric species of *Rodriguezia*). In this circumscription, the genus holds about 130 species, 17 of which are native to Rio Grande do Sul [5]. Of these, only *Gom. hidrophila* Barb. Rodr. and *Gom. barbaceniae* (Lindl.) M. W. Chase & N. H. Williams are terrestrial, while the remaining species are epiphytes. Only *Gom. bifolia* (Sims) M. W. Chase & N. H. Williams and *Gom. paranensoides* M. W. Chase & N. H. Williams reach the Pampa Biome, while the other species occur within the Atlantic Rain Forest Domain. Most yellow, big-flowered species have ornamental appeal (Figure 2) [5,16] and are among the few orchid groups with vernacular names: they are collectively called ‘chuvas de ouro’ or, less commonly, ‘bailarinas’ (‘dancing ladies’) (Figure 2). These species are widely cultivated as species or as hybrids. Notably, *Gomesa flexuosa* (Lodd.) M. W. Chase & N. H. Williams is largely produced in Asiatic countries as a source of cut flowers. Some species, such as *Gomesa concolor* (Hook.) M. W. Chase & N. H. Williams and *Gom. barbata* (Lindl.) M. W. Chase & N. H. Williams, present, in addition to big, relatively long-lived flowers, a remarkable floral variation that could be a valuable source for cultivation (Figure 3).

#### 2.2.1. Current Protection and Threats

As with Cattleya, to date, IUCN has not evaluated any *Gomesa* species native to Rio Grande do Sul on a global scale. The aforementioned SEMAI/RS [10], following IUCN criteria, has proposed a few species as under threat (Summarised in Table 3).

#### 2.2.2. Breeding Systems and Natural Pollination

Breeding systems and pollinators of already studied *Gomesa* spp. are summarised in Table 4. All *Gomesa* spp. studied so far are pollinator-dependent and mostly self-incompatible (Table 4). Natural fruit set is low (≤10.93%) [5].

### 2.3. Chloraeinae Orchids

Although Chloraeinae orchids are, in essence, an Andean group, a remarkable subset of species occurs in southern Brazil and neighbouring regions of Uruguay and Argentina [17,18,19,20]. *Bipinnula*, as a whole, holds about 12 species. While the multiflorous species are restricted to Central Chile, the uniflorous species are a characteristic floristic element of the Pampa Biome and adjacent areas of the Brazilian Mata Atlântica [17,18,19,20]. The five currently accepted uniflorous *Bipinnula* Comm. ex Juss. species are native to Rio Grande do Sul: *Bipinnula biplumata* Rchb.f., *Bipinnula gibertii* Rchb.f., *Bipinnula montana* Arechav., *Bipinnula penicillata* (Rchb.f.) Cisternas & Salazar and *Bipinnula polysyka* Kraenzl. (Figure 4). The exotic flowers of these terrestrial orchids are mostly insect-like in appearance and -except for *B. penicillata*- present fimbriate-pectinate lateral sepals (hence the generic name *Bipinnula*). To the best of our knowledge, *B. polysyka* is endemic to the Pampa biome [18,20], with the remaining species also occurring in grassy-rupicolous areas within the Atlantic Rain Forest domain. To date, *Chloraea membranacea* Lindl. is the sole species in this genus represented in Southern Brazil [4,17] and, consequently, in Rio Grande do Sul. This species occurs in the two Biomes of the State, and its populations, though localised, can be numerous under some circumstances [4,17].

#### 2.3.1. Breeding Systems and Natural Pollination

Published breeding systems of Chloraeinae orchids from Rio Grande do Sul are summarised in Table 5. The two studied species so far are self-compatible and pollinator-dependent [4,19]. Remarkably, the natural fruit set in *Chl. membranacea* is relatively high (46–78%) [4] (Table 5).

#### 2.3.2. Current Protection and Threats

Whereas *B. biplumata*, *B. penicillata* and *C. membranacea* are protected in Municipal and State Parks, respectively, all remaining Chloraeinae species are still not represented in Conservation Units of any kind (Table 6).

### 2.4. Codonorchis canisioi Mansf. (Codonorchideae)

*Codonorchis* Lindl. (Codonorchideae) embraces only two species: the widespread and locally abundant *Codonorchis lessonii* Lindl., from Chilean-Argentinian Patagonia, and *Codonorchis canisioi*, known only from a few localities in Rio Grande do Sul, Brazil [21]. The latter species is extremely rare and was rediscovered in 2018 (Figure 5).

#### 2.4.1. Breeding Systems and Natural Pollination

A comprehensive study of the breeding system of this terrestrial orchid is still lacking because few individuals have been found to date. However, additional populations may still be discovered. Recorded pollinators are bees of Halictidae [21] (Table 7).

#### 2.4.2. Current Protection and Threats

The species has not been evaluated globally, but the SEMAI/RS [10] considered it Critically Endangered (CR) (Table 8). Whereas the type locality of *C. canisioi* is now a private reserve (an RPPN, according to Brazilian nomenclature); this species has not been found there since 1935, even after more recent attempts [21]. The currently known populations occur on private properties subject to fire (a regular practice at the end of the winter and early spring).

### 2.5. Spiranthinae Orchids

Rio Grande do Sul is remarkably rich in terrestrial (rarely epiphytic) Spiranthinae orchids. These species are particularly common in open, grassy areas of either the Pampa or the Mata Atlântica biomes [6]. Still, Rio Grande do Sul harbours a significant diversity of Spiranthinae orchids with colourful flowers and ornamental appeal, especially within the genera *Pachygenium* (Schltr.) Szlach., R.González & Rutk., *Pelexia* Rich., *Pteroglossa* Schltr. *Skeptrostachys* Garay and *Sarcoglottis* C. Presl (Figure 6).

#### 2.5.1. Breeding Systems and Natural Pollination

Breeding systems and pollinators of Spiranthinae species native to Rio Grande do Sul are summarised in Table 9. Overall, the Spiranthinae orchids studied so far are self-compatible and pollinator-dependent (Table 9) [6], with native bees as the main pollinators.

#### 2.5.2. Current Protection and Threats

Few Spiranthinae species native to Rio Grande do Sul have been considered endangered by the SEMAI/RS [10] (see Table 10). In turn, the IUCN has not evaluated any of these globally. We are unaware of the occurrence of either species within any Conservation Units.

### 2.6. Orchidinae Orchids (Habenaria pp.)

Rio Grande do Sul is rich in terrestrial species of the genus *Habenaria* Willd. Those *Habenaria* spp. are mostly common in open, grassy areas of the Pampa or Mata Atlântica biomes [9], and also occur in palustrine or swampy conditions.

#### 2.6.1. Breeding Systems and Pollination

Breeding systems and pollinators of *Habenaria* species native to Rio Grande do Sul are summarised in Table 11. Species studied so far are pollinator-dependent and self-compatible [9]. Moths are the main pollinators (Table 11).

#### 2.6.2. Current Protection and Threats

SEMAI/RS [10] classified five *Habenaria* species as endangered: *Habenaria dutraei* Schltr. (CR), *Habenaria ekmaniana* Kraenzl. (CR), *Habenaria exaltata* Barb. Rodr. (VU), *Habenaria henscheniana* Barb. Rodr. (EN) and *Habenaria pentadactyla* Lindl. (EN). To the best of our knowledge, none of these species is yet represented in Conservation Units. Terrestrial species are threatened by land conversion to agriculture or silviculture. In turn, swamp-dwelling species are threatened by drying of these areas for agricultural purposes.

### 2.7. Seed Measurements, Germination and Conservation

The average half-life (*P*_50_) for *Cattleya* seeds under conventional seed banking (e.g., −20 °C and 5% RH) is about 30 years, with some extending beyond this [22]. Orchid seeds are very small and variable. Size varies among species within the same genus and between genera. Few studies have reported seed size and conservation status for this family [22,23,24,25]. However, orchid seeds may be grouped by clade (Figure 7). Seed size is compared with many orchids here; *Cattleya* orchids form a distinct group, and many have at least medium-lived seeds (*P*_50_ > 25 years) under conservation [22]. For Brazilian tropical terrestrial orchids, little is known about their seeds and conservation. A few contributions describe seed morphology [26] and symbiotic germination, and some have been reported for non-native species [26,27,28]. The same pattern is found for the *Gomesa* group. So far, nothing has been described about seed storability and longevity under conventional seed banking (−20 °C and 5%RH) for this orchid subgroup. As far as we know, ½ [MS] [29] is a good option for *Cattleya* [22,25,30,31,32], for terrestrial species, and for the *Gomesa* group. Nevertheless, seed development must be evaluated. Our team is working to characterise seeds, from linear to volumetric measures, storage reserves, and conservation under conventional seed-banking conditions (e.g., −20 °C and 5% relative humidity), as well as their germination protocols before and after storage [33]. This methodology must be implemented for the former *Sophronitis* Lindl. species, now placed within *Cattleya*, with the smallest seeds for the genus, for the *Gomesa* group and the terrestrials (*Sacoila*, *Habenaria*, and many other Brazilian Orchidoideae).

Further investigations of terrestrial orchids are needed for germination; our first experiments with *Cyclopogon congestus* (Figure 8, Table 12) suggest that terrestrial seeds of this tropical orchid still germinate in the ½ [MS] medium (84.1%). However, in nitrate-rich media, germination occurred without further development, as in temperate terrestrials, as indicated by [34,35]. In nitrate–rich medium (½ [MS]), the oxidation of protocorms is over 40% (Table 12), and in a medium with low nitrate, such as Knudson C [36], germination is very slow, stopping at initial phases of germination even with a large amount in phase 1 (80% germination, Figure 8C, Table 12). In Malmgren medium [37] (no nitrate), germination is high (~95%), and the protocorms still develop rhizoids, indicating seedling development, with no oxidation occurring (95.5% germination and rhizoid development, Figure 8B, Table 12). In the next experiment, we changed the medium, excluding Knudson C medium because it was unresponsive. The germination of three species (Table 13), *Bipinnula montana*, *Sacoila lanceolata*, and *Cyclopogon congestus*, points in the same direction: a nitrate-rich medium (½ [MS]) still supports high germination but not further protocorm growth; the BM1 medium [38] showed promising results, and it is a no-nitrate medium. It is important to know that there is a specific germination response to different media.

## 3. Discussion

### 3.1. General

Among the orchid groups herein considered, 18 endangered species were included, and 11 of them (61.117%) are, so far, likely absent in Conservation Units. Overall, threatened species are concentrated in the genera *Cattleya* (Laeliinae) and *Bipinnula*, which are affected in different ways; the first genus is of ornamental interest (and frequently collected by poachers). In contrast, *Bipinnula* is mostly affected by habitat loss, herbicides, and cattle raising. Only the *Cattleya* species are regularly propagated through commercial, large-scale, and reliable storage and propagation protocols [25,30,32]. Such protocols are largely lacking or still being developed for Oncidiinae and for the terrestrial orchids (Spiranthinae, Codonorchideae, Chloraeinae and Orchidinae). To date, restoration/reintroduction experiments have been applied only to *Cattleya intermedia*, a horticultural species heavily targeted by poachers [13,14,15]. Unfortunately, such trials are completely lacking for the other orchid groups herein considered [13,14,15].

### 3.2. Laeliinae Orchids

Most *Cattleya* species studied so far are consistently pollinator-dependent and self-compatible [7]. This is because, whereas most species can set fruit through self-pollination, animal pollinators are the necessary agents of pollination in the wild. Whereas *Cattleya coccinea* is pollinated by hummingbirds, the other three species rely on native (either solitary or social) bees (Table 2) [7]. Notice, however, that even in self-compatible species, seeds from cross-pollinations may perform at higher fitness, measured by better vigour and higher germination percentages. Within the *Cattleya* species native to Rio Grande do Sul, *Cattleya coccinea* presented higher proportions of embryo-bearing (vs. empty) seeds, suggesting that seeds from cross-pollination may perform better during germination. This, however, has not yet been tested experimentally [7]. Both studied *Epidendrum* species are pollinator-dependent and rely on diurnal Lepidoptera as pollinators [8,11]. However, whereas *E. fulgens* ranks as self-compatible [8], *E. densiflorum* is self-incompatible [11]. Remarkably, all Laeliinae species studied so far showed low (≤14.2%) fruiting success (Table 2). This pattern seems fully consistent with the flowers being nectarless, which prompts few or scarce pollinator visits [7]. With the notable exception of *C. coccinea*, all other *Cattleya* species have been placed under a category of endangerment. Fortunately, all mentioned *Cattleya* species are protected in Conservation Units (either National or State Parks). However, *C. intermedia* and *C. tigrina* are targets of poachers and are regularly seen being sold at street markets in Rio Grande do Sul, along with other ornamental Laeliinae orchids such as *Brassavola tuberculata* Hook. and *E. fulgens*. *Cattleya* species are among the most traded ornamental orchids worldwide. Researchers have studied seed behaviour in detail under different sowing and storage conditions. Consequently, all *Cattleya* species from Rio Grande do Sul are regularly propagated for commercial purposes using reliable, well-established protocols [22,25,30,32]. Successful reintroduction trials have been conducted in Rio Grande do Sul for *Cattleya intermedia* [13,14,15], but are lacking for the other considered species. Overall, luminosity affected plant survival [13], and plants reintroduced in the forest interior and under the canopy experienced less herbivory than those reintroduced at the forest edge [13,15]. Some plants reintroduced to the canopy flowered after three years [15]. So far, plant growth has not been linked to the pH and wood thickness of the plant hosts (trees where the orchids are growing) [15]. Among all orchid groups considered in this contribution, *Cattleya intermedia* is the only one for which such trials have been applied. To date, no *Epidendrum* species native to Rio Grande do Sul is considered endangered [10], but -as said above-, *Epi. fulgens* is frequently collected by poachers and sold. *Epidendrum fulgens* is widespread, forming large populations, even in anthropic conditions, such as *Pinus* and *Eucalyptus* plantations, so that poaching, for a while, does not seem a cogent risk for this orchid.

### 3.3. Oncidiinae Orchids

Most *Gomesa* species studied so far are consistently pollinator-dependent (Table 4) but, unlike Laeliinae orchids, they are fully or mostly self-incompatible [5]. This is because, beyond relying on animal pollinators, they need pollen flow among conspecific individuals for successful pollination, making them particularly fragile in contexts of either habitat loss or poaching. Unlike self-compatible orchids that could -hypothetically- recolonise an area through a single remnant individual, self-incompatible orchids compulsorily need the presence of at least a few different individuals coupled with the presence and activity of their pollinators. They need cross-pollination to set fruit and produce viable seed [5]. A summary of the breeding system and known pollinators of *Gomesa* spp. native to Rio Grande do Sul is presented in Table 4. Notably, all species whose pollination could be documented in detail are pollinated by females of native, solitary, oil-gathering bees of the genera *Centris* (*Gom. bifolia*, *Gom. flexuosa*), *Paratetrapedia* (*Gom. cornigera*), *Trigonopedia* (*Gom. cornigera*, *Gom. ranifera*), *Lophopedia* and *Arhysoceble* (*Gom. riograndensis*) [5]. All these native bees need conserved areas to thrive and nest, so for *Gomesa* populations to endure, not only must a minimum number of individuals persist (to circumvent self-incompatibility), but their respective pollinators must also be present and resilient [5]. To the best of our knowledge, except for *Gom. venusta* (Drapiez) M. W. Chase & N. H. Williams; all remaining *Gomesa* species native to Rio Grande do Sul are represented in Conservation Units (either Federal, State, or Municipal Parks). However, *Gom. flexuosa*, *Gom. uniflora* (Booth ex Lindl.) M. W. Chase & N. H. Williams, and, to a lesser extent, *Gom. barbata* are regularly collected by poachers and often sold at street markets in Rio Grande do Sul, along with other Laeliinae orchids already mentioned. Whereas the ‘dancing ladies’ are undoubtedly of ornamental appeal and relevance, they are not as well studied as *Cattleya* regarding their in vitro propagation [39,40,41,42,43]. Some commercial hybrids are produced on a large scale, but protocols for most natural species remain to be established. We are unaware of any reintroduction efforts, either in Rio Grande do Sul or elsewhere in Brazil.

### 3.4. Chloraeinae Orchids

A comprehensive study on the breeding system and pollination biology of all uniflorous *Bipinnula* species is in progress and is expected to be published soon (Agustín Sanguinetti and Cristiano Buzatto, pers. comm., in September 2026). In contrast, information on the pollination needs of *Chloraea membranacea* has been published. A study involving populations from Rio Grande do Sul and Buenos Aires province (Argentina) indicates that plants are consistently self-compatible, yet pollinator-dependent [4]. The main pollinators are solitary bees of the family Halictidae, which are deceived because the flowers offer no reward. The fruiting success is relatively high for a terrestrial orchid [4] (Table 5). *Bipinnula penicillata’s* pollination has been studied in Argentina [19]. Male *Campsomeris* wasps that attempt copulation with the flowers and the plants are likely self-compatible [19]. Remarkably, *B. giberti* and *B. polysyka* are recent additions to the orchid Flora of Rio Grande do Sul [18,20]. Globally, only *B. polysyka* has been evaluated under IUCN guidelines and, remarkably, classified as DD (data deficient), illustrating the scarcity of information on these terrestrial orchids. On the other hand, Rio Grande do Sul [10] has placed all remaining Chloraeinae species under some threat category (Table 6). *Bipinnula montana* and *Chl. membranacea* received a VU category [10], consistent with their localised yet locally abundant populations. Whereas all Chloraeinae orchids from RS suffer moderate poaching, the main threats to these species come from habitat loss and some vegetation maintenance practices: some populations are being threatened by the systematic use of glyphosate and other herbicides to keep the roadsides clean, and we have seen important populations of *B. montana* being accidentally depleted in this way in recent years (R. Singer and R. Farias-Singer, pers obs). For Orchidoideae, information on propagating Chilean Chloraeinae species is relatively abundant [44,45,46,47,48], but information on non-Chilean species is still under development. Some experiments on propagating *B. penicillata* and *C. membranacea* have been developed in Argentina [44]. Protocols for *Bipinnula* species originating from Rio Grande do Sul are currently under development. *Chloraea membranacea* and some *Bipinnula* species are already being cultivated ex situ at the Porto Alegre Botanical Garden. These plants are mostly being rescued from places where either native vegetation is being suppressed or, as commented above, threatened with herbicides. When in bloom, these plants are being used to produce fruit whose seeds will be used in germination protocols.

### 3.5. Codonorchideae

Until 2018, *C. canisioi* was only known from the type locality. However, this tiny terrestrial orchid wasn’t recorded at the type locality since 1935 [21]. In 2018, two small populations were reported, and the plant could be illustrated, since the original description lacked illustrations. In addition, some features (ex: the fruit) lacking in the original description were presented. According to the most recent observations, flowering and fruiting proceed very quickly (within a few weeks) [21]. After that, the plants lose their leaves completely, and only some roundish tubers remain underground. Thus, it seems very possible that other populations are still being overlooked. Therefore, a considerable effort to locate additional populations seems imperative. Fortunately, researchers found two small populations in other localities, about 200 km from the type locality [21]. SEMAI/RS [10] considered it CR, following the IUCN criteria (Table 8), but a global evaluation is still lacking. As noted above, the plants are small and inconspicuous, and we consider that other populations may still exist in Rio Grande do Sul or in the neighbouring State of Santa Catarina. However, an intensive search for these putative overlooked populations is yet to be performed. Because the species is rare, no propagation and/or reintroduction protocols exist, and it seems unlikely that such studies have started.

### 3.6. Spiranthinae Orchids

Overall, few Spiranthinae orchids are regularly cultivated. Of the species native to Rio Grande do Sul, only *Sacoila lanceolata* (Aubl.) Garay (Figure 6) is seen with some frequency in cultivation, but this showy orchid is still not regularly propagated or available in trade. Spiranthinae orchids either from Rio Grande do Sul or other Brazilian States are consistently self-compatible, but mostly pollinator-dependent (Table 9). Capsule development is rapid, and seeds are normally released within a month after pollination [6]. Except for *S. lanceolata*, which is pollinated by hummingbirds, all other Spiranthinae species studied so far are insect-pollinated, especially by native bees [6]. When quantified, reproductive success in Nature is high (≥22%) (Table 9). Among Spiranthinae orchids native to Rio Grande do Sul, only *S. lanceolata* is cultivated with some frequency. Information is scattered for some species, but fully functional protocols are still under development. We are unaware of any attempts or protocols to reintroduce these species. Few Spiranthinae species have been considered endangered in Rio Grande do Sul [10]. *Pelexia ekmanii* (Krzl.) Schltr. has been mainly recorded in Northeastern Rio Grande do Sul, a region extensively devoted to soybean plantations. On the other hand, *Sarcoglottis uliginosa* (Barb. Rodr.) Barb. Rodr. exclusively lives in swamps that are often dried for agricultural purposes (Cristiano Buzatto, pers. comm.). To the best of our knowledge, we are unaware of both species occurring within any Conservation Units.

### 3.7. Orchidinae Orchids

Several species present nocturnally fragrant, big, white-flowered species that have considerable ornamental appeal (*Habenaria macronectar* (Vell.) Hoehne, *Habenaria gourlieana* Gillies ex Lindl. and similar species) [9]. However, most of these species dwell in swamps, a difficult condition to replicate under cultivation. Some terrestrial species such as *Habenaria josephensis* Barb. Rodr. and *Habenaria pleiophylla* Hoehne & Schltr. are cultivated in particular collections. All *Habenaria* spp. studied so far are pollinator-dependent, self-compatible and mostly pollinated by nocturnal Lepidoptera [9]. Notably, the showy flowers of *Habenaria montevidensis* Lindl. are pollinated by diurnal butterflies instead [9]. Table 11 summarises knowledge of the pollination and breeding systems of *Habenaria* spp. native to Rio Grande do Sul. Natural fruiting success is high (≥69%) (Table 11), in agreement with the presence of nectar in the flowers as well as with the uniformity of self-compatibility within the studied species. Remarkably, some species can thrive under anthropic conditions: the palustrine *Habenaria repens* Nutt. can quickly colonise ponds or small lakes when surrounded by other aquatic plants. The terrestrial *H. josephensis* is frequent in the understorey of *Pinus* or *Eucalyptus* plantations, often near other native terrestrial orchids, such as *Liparis nervosa* and *Epidendrum fulgens*. Only a few *Habenaria* species native to Rio Grande do Sul are cultivated, and the showiest-flowered ones require aquatic conditions that are difficult to replicate. In general, *Habenaria* seeds have a shell that makes germination under laboratory conditions very difficult. Information on vegetative propagation is scattered for some species (e.g., *Habenaria bractescens* Lindl.), but fully functional protocols for seed storage and germination are still to be developed [49,50,51]. We are unaware of any attempts or protocols to reintroduce *Habenaria* species in Brazil.

## 4. Materials and Methods

All the available information pertinent to the ex-situ and in situ conservation of Orchidaceae native to Rio Grande do Sul was retrieved from the three major databases: Web of Science, Scopus, and Google Scholar. Coverage included natural pollination, pollinators, fruiting success, breeding systems, propagation and storage protocols, and reintroduction/restoration protocols; searches concluded in early August 2026. Only published information, in the form of articles, books and/or book chapters, is considered. Symposium abstracts are not considered here, primarily because they are not peer-reviewed data sources. Plant names follow IPNI (International Plant Name Index). Plant distribution to ascertain occurrence within conservation Units was checked through the Species List database at CRIA (Campinas, São Paulo, Brazil), which hosts the larger platform, including Brazilian Herbaria (which, in turn, include herbaria from Rio Grande do Sul). This information is complemented with our own field data. Illustrations were prepared from our own photographs, mounted as LCDP (Lankester Composite Digital Plates), following the methodologies described by Pupulin and Bogarin [52] and Singer et al. [53]. This contribution does not embrace all threatened orchid species in Rio Grande do Sul [10]. Instead, it focuses on orchid groups with ornamental potential or that are already traded (see Table 1, Table 3, Table 6, Table 8 and Table 10).

### 4.1. Seed Measurements

All seeds from RS and the others were in the UNOESTE Orchid Seedbank. Seed morphology was obtained by microscopic measurement, as in Fileti et al. [25]. Seeds were obtained from collections or by cross-pollination; UFRGS provided the *Gomesa/Trichocentrum* terrestrials. *Disa uniflora*, *Sacoila lanceolata*, *Prescottia stachyoides*, *Eltroplectris triloba,* and *Cattleya* were from the Orchid Seedbank of UNOESTE. The capsules collected at the beginning of dehiscence were dried in a saturated lithium chloride solution [54] for one week. The seeds were removed from the capsules, cleaned, inserted into a plastic vial with a silicone seal O-ring, and stored in a freezer over silica gel at −18 °C. All species were collected in more than three capsules from different plants. The seeds, at least 40, were placed on a microscope slide, and images were obtained under a binocular microscope (OPTON TIM-2T, ANATOMIC, Cotia, Brazil) coupled to a digital camera (OPTON 3680, ANATOMIC, Cotia, Brazil) and connected to a computer system (Lenovo loaded with Windows 7). Seed sizes were measured using ISCapture software (version 3.7, Fuzhou Tucsen Photonics Co., Ltd., Fuzhou, China). The length and the width of individual seeds were taken separately, and the embryo volume was calculated as a prolate spheroid:

$$ev\;=\;4.19ab^{2}$$

where *ev* is the volume of the prolate spheroid embryo, *a* is the length of the longest semi-axis, from tip to tip in the largest way, and *b* is the length of the shortest semi-axis. Seed volume was calculated as a cone multiplied by two:

$$sv\;=\;2.(\frac{1}{3}.\pi .r^{2}.h)$$

where *h* is the height and *r* is the radius of the cone. The air space was calculated by subtracting the embryo volume (*ev*) from the seed volume. The ratio of embryo to seed volume was calculated by dividing the embryo volume by the seed volume. The following species were included in this part: *Bipinnula gibertii*, *Bipinnula montana*, *Bipinnula polysyka*, *C. cernua*, *C. intermedia*, *C. purpurata*, *C. tigrina*, *Chloraea membranacea*, *Cyclopogon congestus*, *Cyclopogon elegans*, *Disa uniflora*, *Eltroplectris triloba*, *Gom concolor*, *Gom flexuosa*, *Gom paranensoides*, *Gom varicosa*, *Habenaria bractescens*, *Habenaria repens*, *Prescottia stachyoides*, *Sacoila lanceolata*, *Sarcoglotis ventricosa*, *Sauroglossum elatum*, *Skept arechavalentanii*, *Trichocentrum pumilum.* The South African species *Disa uniflora* was included in the phenetic clustering and ordination analyses as a reference taxon rather than a functional outgroup, given the unrooted nature of the distance-based algorithms. Accordingly, *Prescottia* and *Eltroplectris* (originating from São Paulo) were retained as reference taxa for Cranichidae, whereas the *Cattleya* group was excluded as it did not fulfill this comparative requirement. *Disa uniflora*, *Prescottia stachyoides*, and *Eltroplectris triloba* were included as reference taxa for terrestrials.

The seed data were analysed by ANOVA, and when significant, Tukey’s HSD test was applied using SISVAR (6.6 Build: 106, [55]); the full dataset was then analysed by UPGMA with Gower similarity index using the PAST software 5.3 [56].

### 4.2. Seed Germination

Two experiments were conducted with the terrestrial samples. In both samples, near 5 mg was inserted into a syringe, imbibed in 10% sucrose solution for 24 h, kept at 25 °C, washed twice in water, and disinfected with sodium dichloroisocyanurate (3 g L^−1^) with 100 µL L^−1^ Tween 80 for ten minutes, washed twice in distilled autoclaved water, and sown in a laminar flow hood [57]. For the first one, only *Cyclopogon congestus* was used in three different media: Malmgren [37], ½ MS [29], and Knudson C [36], with three replicates per medium, with three marked fields in each. The plates were maintained in the dark, at 25 °C, and scored biweekly until 10 weeks. For the second experiment, seeds of *Sacoila lanceolata*, *Bipinnula montana*, and *Cyclopogon congestus* were dispersed in each of the following media: Malmgren [37], ½ MS [29], a modification of ½ [MS] without nitrate, containing 170 mg L^−1^ urea, 790 mg L^−1^ potassium chloride, and 170 mg L^−1^ potassium phosphate monobasic to adjust the potassium and phosphate supply, and BM1 media [58]. In vitro germination tests used three replicates (Petri dishes) per treatment, with approximately 150 seeds scored per dish. The data were transformed by $y=\arcsin x^{-\frac{1}{2}}.$ Data were subjected to Analysis of Variance (ANOVA) followed by Tukey’s post-hoc test (*p* < 0.05). All chemical were purchased from Sigma-Aldrich Co. (Saint Louis, MO, USA). Given the exploratory nature of the culture media evaluation, these comparisons are preliminary observations.

## 5. Conclusions

Owing to the patterns arising from this contribution, we would like to recommend the following:

(1) A considerable investment in extensive fieldwork to locate eventually overlooked populations, especially for as-yet threatened species.

(2) Whenever possible, the creation of Conservation Units, especially for endangered species not yet protected in situ.

(3) Promoting ex situ protection in the form of specimens cultivated in Botanical Gardens or analogue collections, coupled with the storage of seeds and/or pollinia in germplasm banks.

(4) Encouraging local researchers to propagate native species and perform reintroduction/restoration experiments, if possible, within Conservation Units.

## Figures and Tables

**Figure 1:**
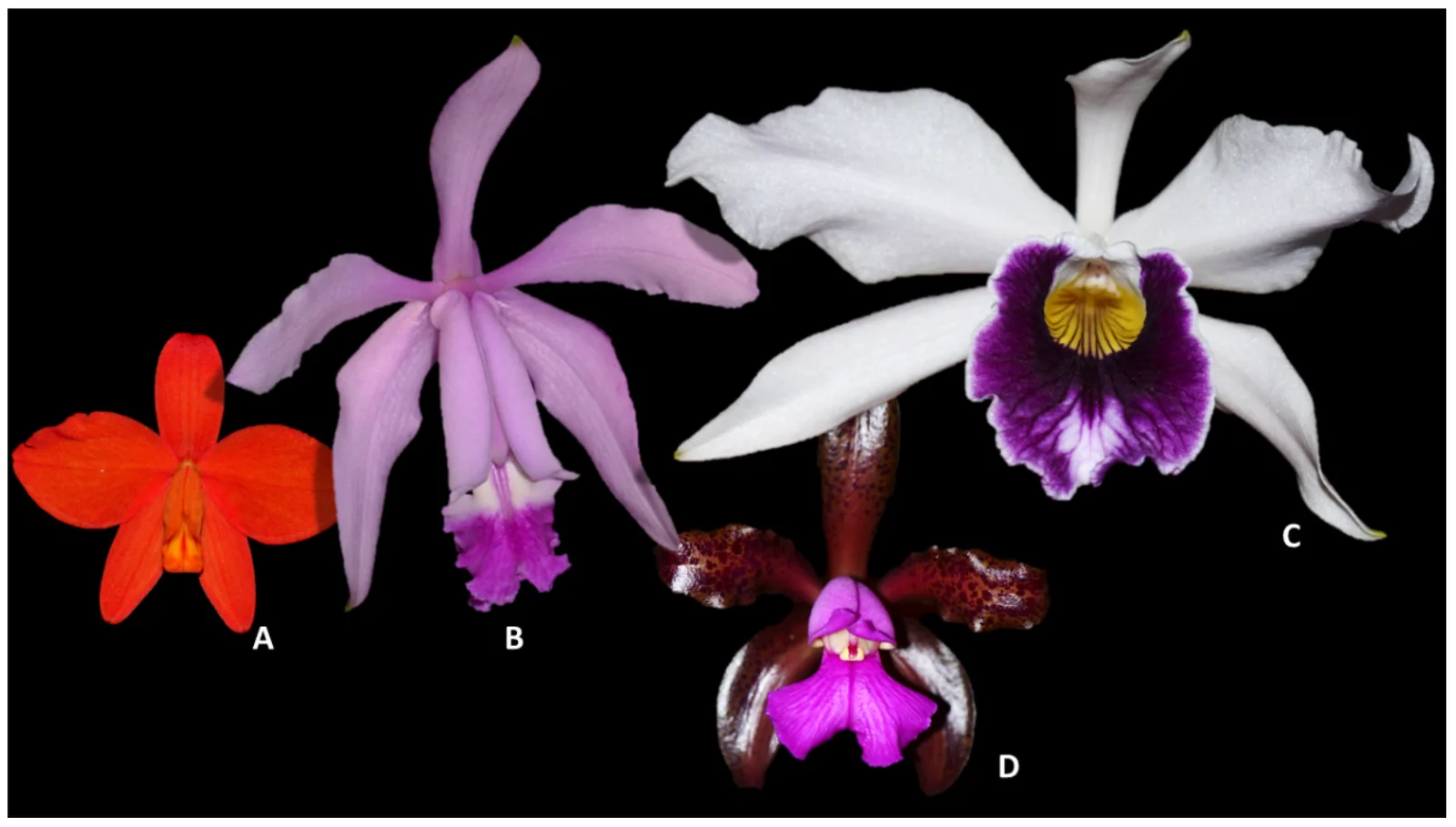
*Cattleya* species native to Rio Grande do Sul State. (**A**) *Cattleya coccinea* (**B**), *C. intermedia* (**C**), *C. purpurata* (**D**), *C. tigrina*. Abbreviations of scientific names followed the rules of the Royal Horticultural Society (for orchid genera and hybrids (RHS 2017).

**Figure 2:**
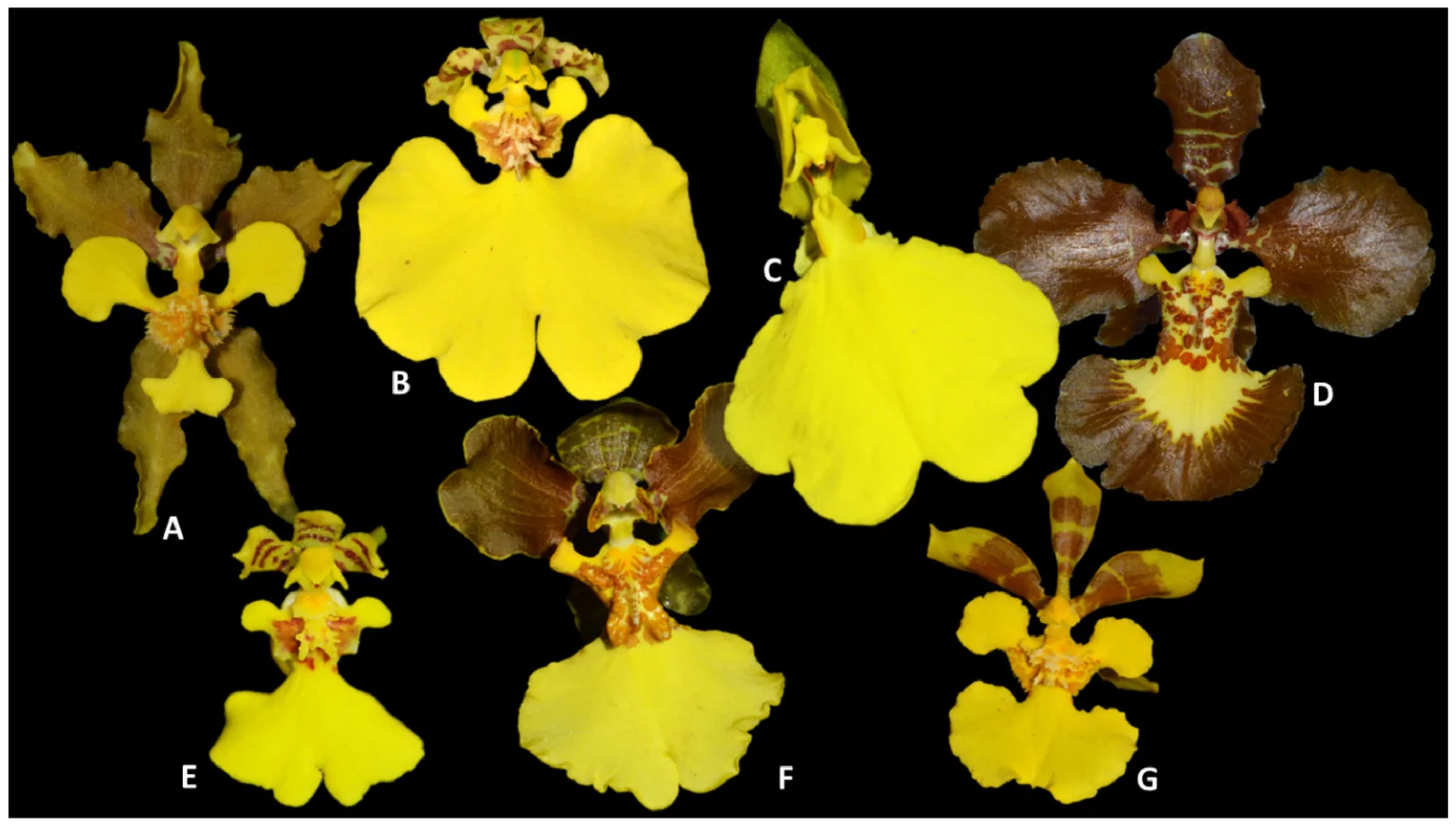
*Gomesa* species native to Rio Grande do Sul State: (**A**) *Gom. barbata*, (**B**) *Gom. bifolia.* (**C**) *Gom. concolor*; (**D**) *Gom. imperatoris-maximilliani;* (**E**) *Gom. flexuosa.* (**F**) *Gom. pectoralis*, and (**G**) *Gom. uniflora*.

**Figure 3:**
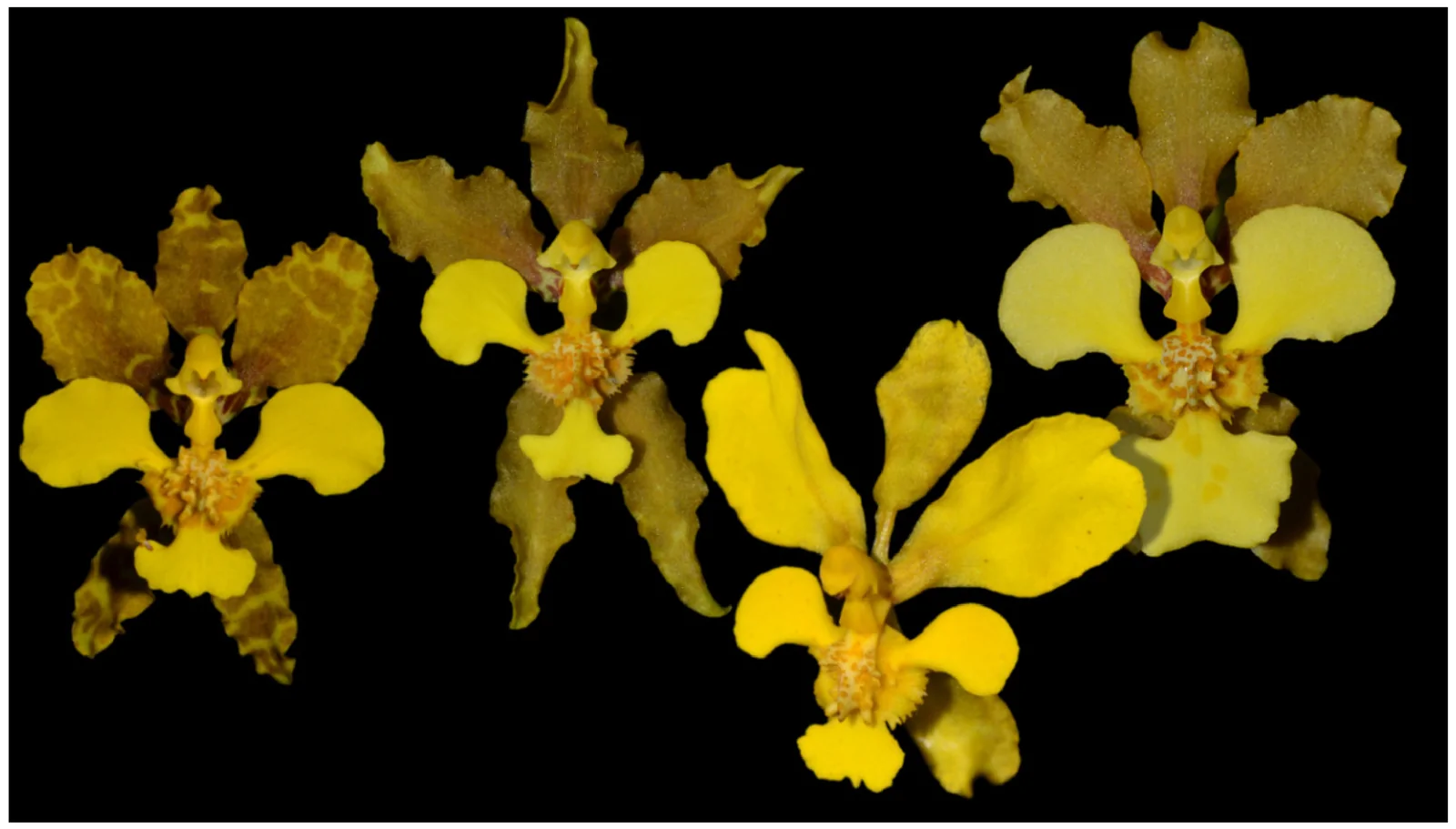
Floral variation in specimens of *Gomesa barbata* from different populations in Rio Grande do Sul State.

**Figure 4:**
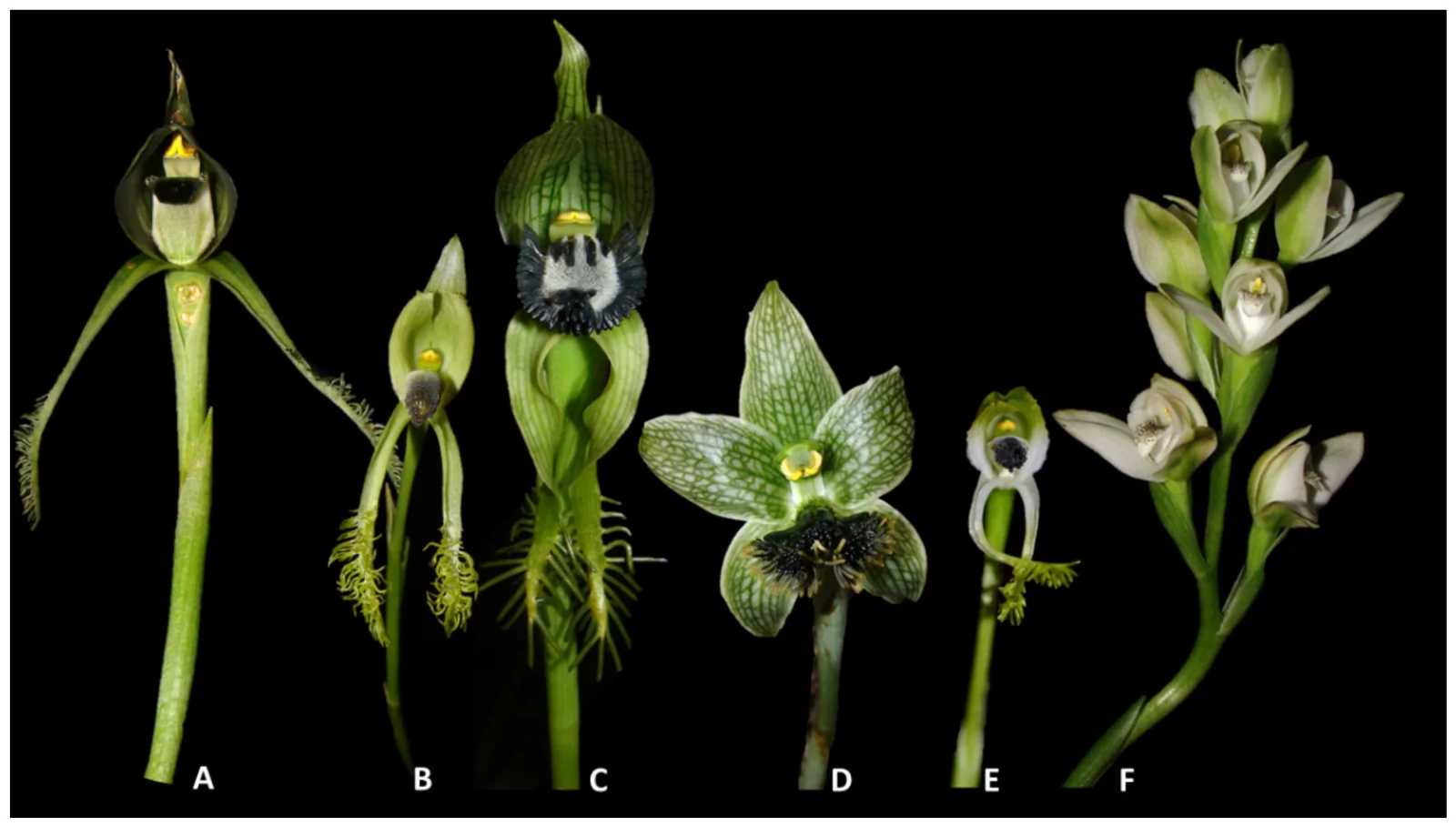
Chloraeinae orchids native to Rio Grande do Sul. (**A**) *Bipinnula biplumata.* (**B**) *B. giberti.* (**C**) *B. montana.* (**D**) *B. penicillata.* (**E**) *B. polysyka*. (**F**) *Chloraea membranacea*.

**Figure 5:**
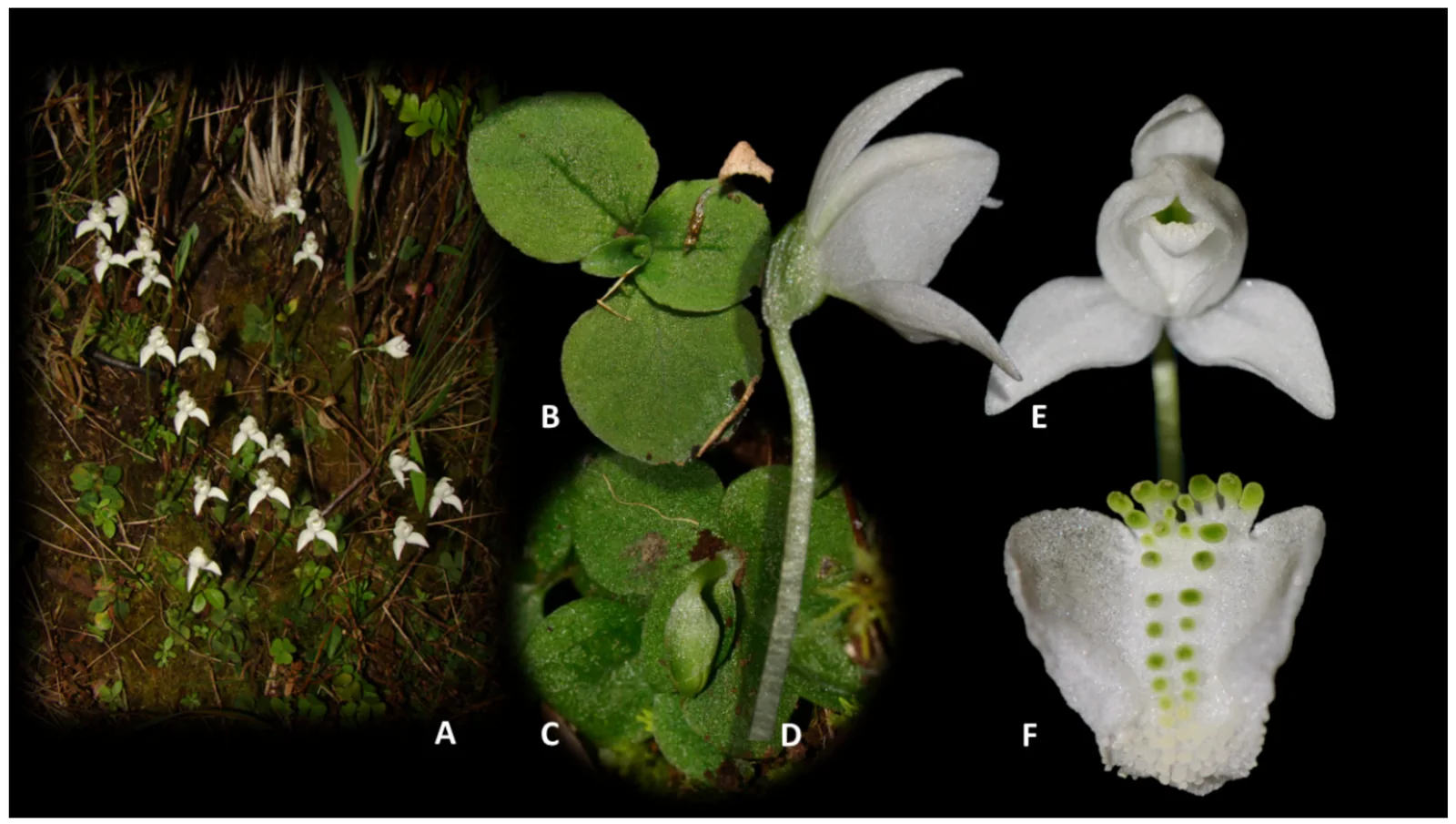
*Codonorchis canisioi* (Codonorchideae), vegetative and floral features. (**A**) Plant group in the field. (**B**) Leaf rosette. (**C**) Developing bud. (**D**) Inflorescence in lateral view. (**E**) Flower in frontal view. (**F**) Labellum.

**Figure 6:**
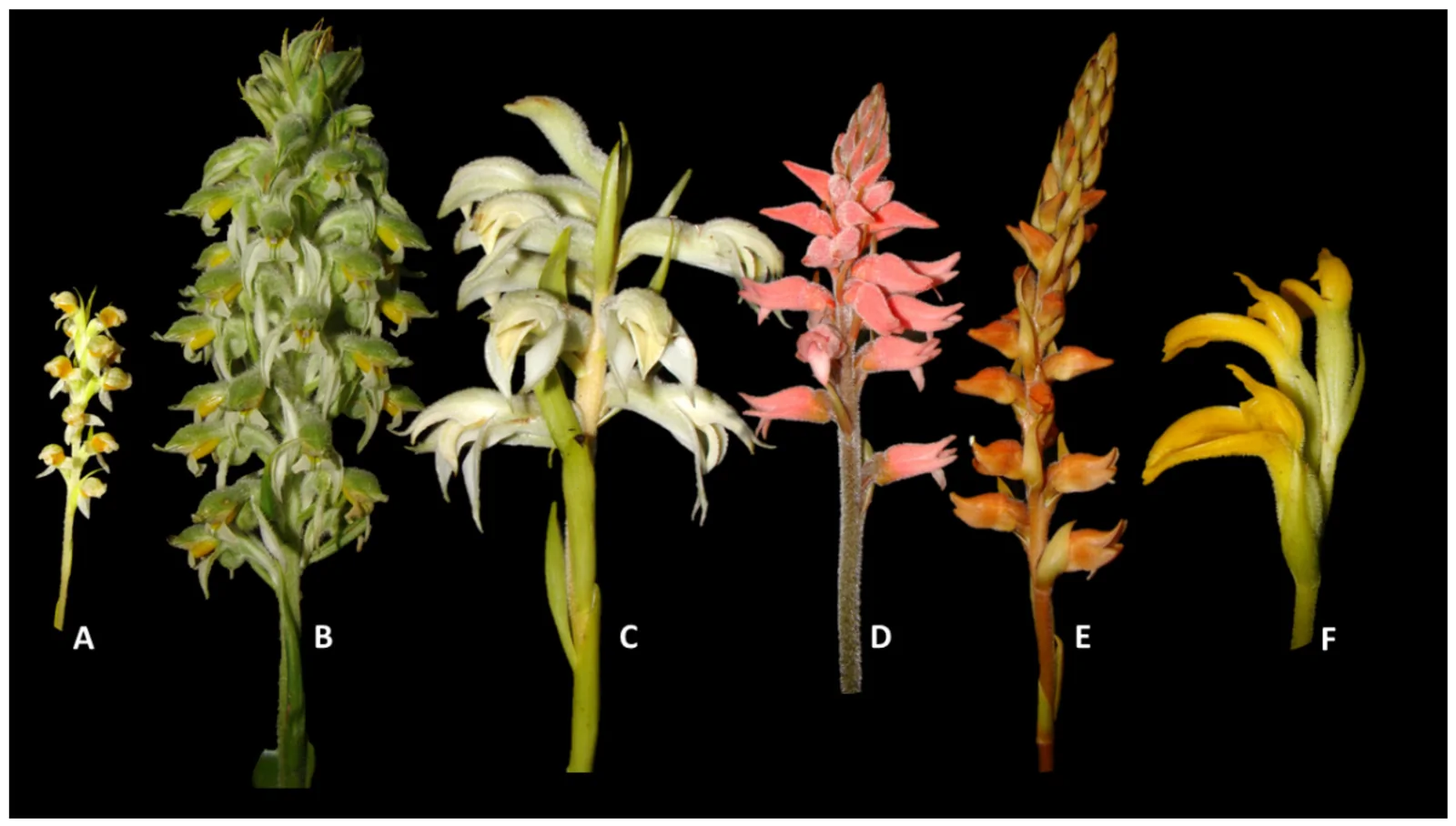
Diversity of floral features in Spiranthinae orchids native to Rio Grande do Sul. (**A**) *Brachystele subfiliformis*. (**B**) *Pachygenium pteryganthum*. (**C**) *Pteroglossa macranta*. (**D**) *Sacoila lanceolata*. (**E**) *Skeptrostachys arechavaletai*. (**F**) *Sarcoglottis uliginosa*.

**Figure 7:**
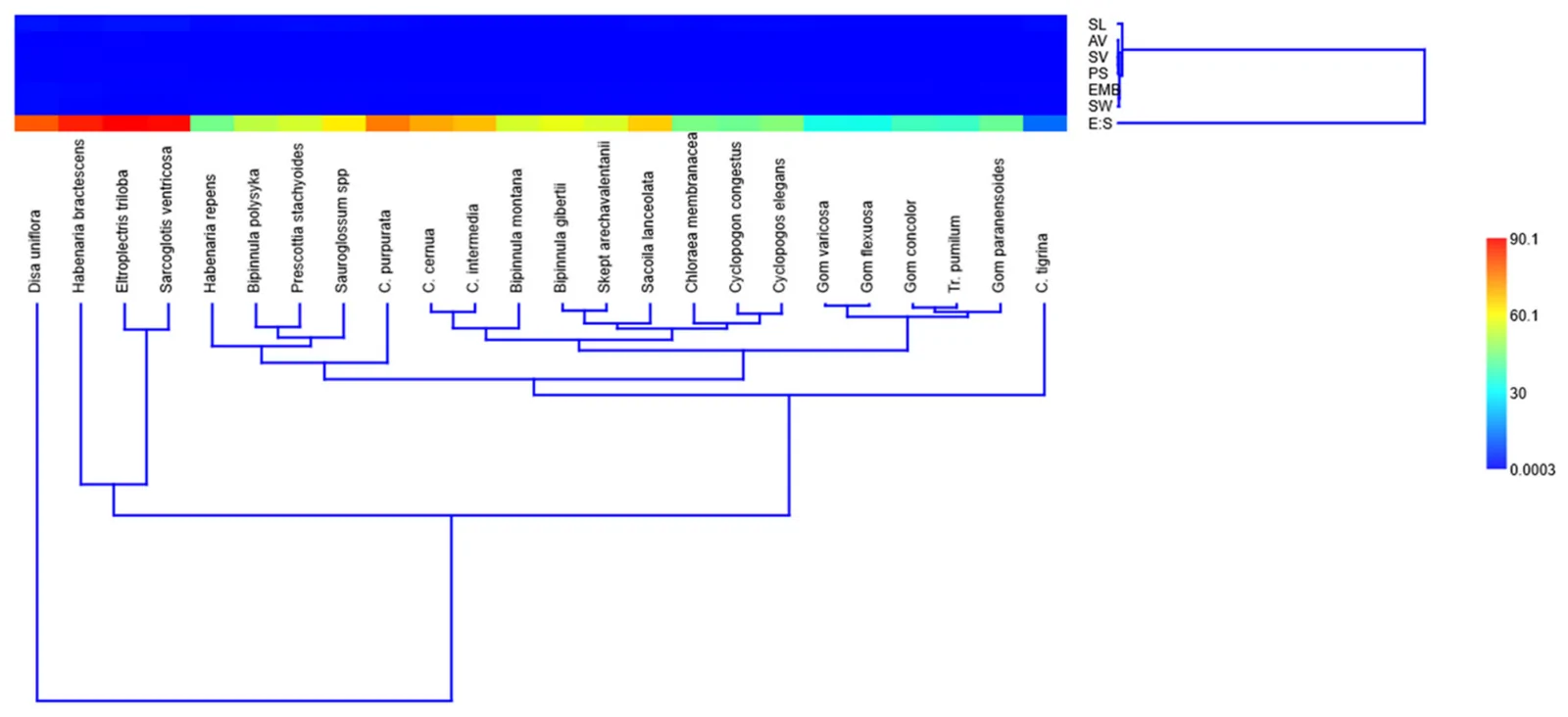
Hierarchical tree based on seed linear and volumetric measurements, using the UPGMA algorithm with Gower similarity index. Terrestrial (Orchidoideae) and Epiphytic *Gomesa* form distinct groups (number of seeds (n) = 40 for all species). SL—Seed length; AV—air volume, SV—seed volume, PS—prolate spheroid, EMB—embryo width, SW—seed width, E:S—embryo: seed volume relation. Colour scale: red—highest values; cold blue lower values.

**Figure 8:**
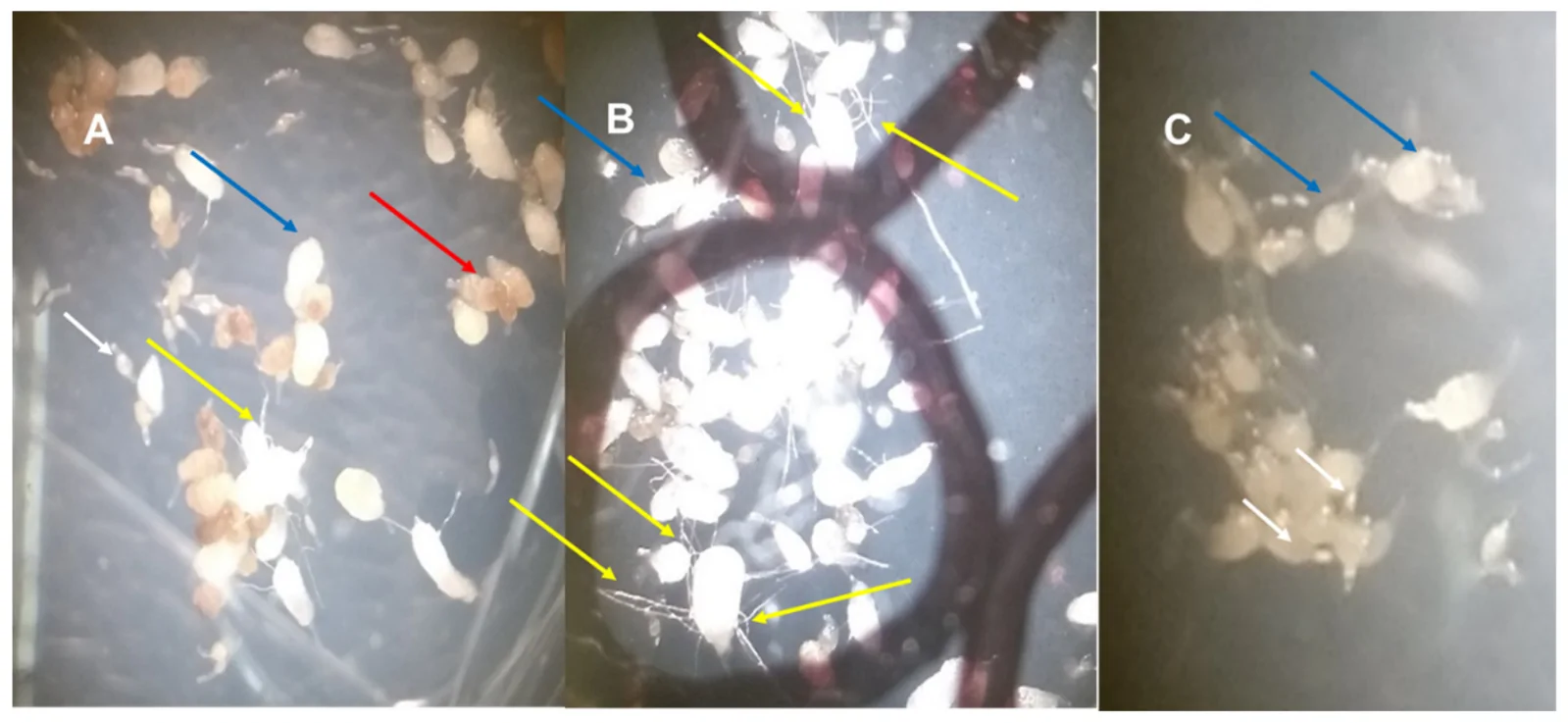
Germination of *Cyclopogon congestus* in three different culture media: (**A**) Murashige and Skoog at half the nutrient concentration, (**B**) Malmgren, and (**C**) Knudson C. Blue arrows indicate germinated seeds, red arrows oxidised seeds, white arrows non-germinated seeds and yellow arrows are protocorms with rhizoids. (n = 150 per replication).

**Table 1:** Conservation status of *Cattleya* species native to Rio Grande do Sul.

Orchid Species	IUCN (Global)	SEMAI/RS	Represented in Conservation Units?
*Cattleya coccinea*	NE	---	Federal and State Parks
*Cattleya intermedia*	NE	VU	Federal and State Parks
*Cattleya purpurata*	NE	EN (as *Brasilaelia purpurata*)	State Parks
*Cattleya tigrina*	NE	EN	State Parks
*Epidendrum densiflorum*	NE	---	National and State Parks
*Epidendrum fulgens*	NE	---	National, State and Municipal Parks

NE: not evaluated.

**Table 2:** Breeding systems and pollinators of *Cattleya* species native to Rio Grande do Sul [7,8,11].

Orchid Species	Breeding System	Pollinators	Natural Fruit Set
*Cattleya coccinea*	Self-compatible (*)	Hummingbirds	4.5–6.7%
*Cattleya intermedia*	Self-compatible	Native Bumblebees, Carpenter bees	1.5–5.4%
*Cattleya purpurata*	Self-compatible	Solitary native bees, Native Bumblebees	0–1.3%
*Cattleya tigrina*	Self-compatible	Carpenter bees	0.51–14.2%
*Epidendrum densiflorum*	**Self-incompatible**	Diurnal Lepidoptera	2.85%
*Epidendrum fulgens*	Self-compatible	Diurnal Lepidoptera	---

(*): fruits from cross-pollinations yielded higher percentages of seeds with embryos.

**Table 3:** Conservation status of threatened *Gomesa* species native to Rio Grande do Sul.

Orchid Species	IUCN (Global)	SEMAI/RS	Represented in Conservation Units?
*Gom. riograndensis*	NE	VU (as *Baptistonia riograndensis*)	State Parks
*Gom. venusta*	NE	CR (as *Baptistonia venusta*)	Apparently not

NE: not evaluated.

**Table 4:** Breeding systems and pollinators of *Gomesa* species native to Rio Grande do Sul, according to [5]. *: data presented in the Supplementary Materials of [5].

Orchid Species	Breeding System	Pollinators	Natural Fruit Set
*Gom. cornigera*	Self-incompatible	Oil-collecting bees	≤5.33%
*Gom. flexuosa*	Self-incompatible	Oil-collecting bees	≤5.6%
*Gom. ranifera*	Self-incompatible	Oil-collecting bees	≤10.93%
*Gom. riograndensis*	Partially self-compatible	Oil-collecting bees	≤5.42%
*Gom. Barbata* *	Partially self-compatible	Unknown	Unknown
*Gom. concolor* *	Self-incompatible	Unknown	Unknown
*Gom. Gomezoides* *	Self-incompatible	Unknown	Unknown
*Gom. imperatoris-maximiliani* *	Self-incompatible	Unknown	Unknown

**Table 5:** Known breeding systems and pollinators of Chloraeinae species native to Rio Grande do Sul [4,19].

Orchid Species	Breeding System	Pollinators	Natural Fruit Set
*Bipinnula penicillata*	Self-compatible	*Campsomeris* wasps	
*Chloraea membranacea*	Self-compatible	Halictidae bees	46–78%

**Table 6:** Conservation status of Chloraeinae orchids native to Rio Grande do Sul.

Orchid Species	IUCN (Global)	SEMAI/RS	Represented in Conservation Units?
*Bipinnula biplumata*	NE	CR	Municipal
*Bipinnula giberti*	NE	CR	No
*Bipinnula montana*	NE	VU	No
*Bipinnula penicillata*	NE	EN	Municipal
*Bipinnula polysyka*	DD	---	No
*Chloraea membranacea*	NE	VU	State Parks

NE: not evaluated. DD: data deficient–Because of its recent incorporation to the Brazilian Flora no risk assessment was made for *B. polysyka* as yet.

**Table 7:** Available information for the reproduction of *Codonorchis canisioi* (Codonorchideae), according to [18].

Orchid Species	Breeding System	Pollinators	Natural Fruit Set
*Codonorchis canisioi*	Unknown	Halictidae bees	Unknown

**Table 8:** Conservation status of *Codonorchis canisioi* in Rio Grande do Sul.

Orchid Species	IUCN (Global)	SEMAI/RS	Represented in Conservation Units?
*Codonorchis canisioi*	NE	CR	Yes (Type locality is a private reserve, but not seen since 1935). *

NE: not evaluated. *: Recently found populations in private properties only.

**Table 9:** Breeding systems and pollinators of Spiranthinae species native to Rio Grande do Sul, according to [6].

Orchid Species	Breeding System	Pollinators	Natural Fruit Set
*Brachystele camporum* Schltr.	Self-compatible	Honeybees, Halictidae	---
*Brachystele dilatata* Schltr.	Self-compatible	Unknown	---
*Cyclopogon argyrifolius* (Barb.Rodr.) Szlach. & Rutk.	Self-compatible	Unknown	---
*Cyclopogon congestus* (Vell.) Hoehne	Self-compatible	Halictidae bees	---
*Cyclopogon elegans* Hoehne	Self-compatible	Halictidae bees	---
*Mesadenella cuspidata* (Lindl.) Garay	Self-compatible	Probably Halictidae bees	---
*Pachygenium bonariense* (Lindl.) Szlach., R.González & Rutk	Self-compatible	Bumble-bees	89–90%
*Pachygenium pteryganthum* (Rchb.f. & Warm.) Szlach.	Self-compatible	*Centris* bees	22–50%
*Sacoila lanceolata*	Self-compatible	Hummingbirds	83.2%
*Sarcoglottis ventricosa* (Vell.) Hoehne	Self-compatible	Probably Euglossine bees	---
*Sauroglossum elatum* Ames	Self-compatible	Moths	33.4%

**Table 10:** Spiranthinae orchids in Rio Grande do Sul threatened with extinction.

Orchid Species	IUCN (Global)	SEMAI/RS [12]	Represented in Conservation Units?
*Pelexia ekmanii*	NE	EN	Unknown
*Sarcoglottis uliginosa*	NE	EN	Unknown

NE: not evaluated.

**Table 11:** Breeding systems and pollinators of *Habenaria* species native to Rio Grande do Sul, according to [9].

Orchid Species	Breeding System	Pollinators	Natural Fruit Set
*Habenaria johannensis*	Self-compatible	Sphingidae moths	92–97% (2 years)
*Habenaria macronectar*	Self-compatible	Sphingidae moths	69–86% (2 years)
*Habenaria megapotamenis*	Self-compatible	Sphingidae moths	84–92% (2 years)
*Habenaria montevidensis*	Self-compatible	Butterflies (Skippers)	78.32%

**Table 12:** Germination, rhizoid development and oxidation percentage of *Cyclopogon congestus* seed in three different media: ½ [MS], Malmgren and Knudson C.

	*Cyclopogon congestus*		
	Germination	Rhizoid	Protocorm Oxidation
	(%)		
½ [MS]	85 b	7.7 b	56 a
Malmgren	95 a	36.3 a	2.7 b
Knudson C	80 b	0 b	1.3 b

Different letters in the column indicate significant differences by Tukey’s Test (*p* < 0.05).

**Table 13:** Initial germination of seeds from cross-pollination of *Bipinnula montana*, *Sacoila lanceolata* and *Cyclopogon congestus* in four different media: ½ [MS], ½ [MS] without nitrates, BM1 and Malmgren.

Germination (%)			
Media	*Sacoila lanceolata*	*Bipinnula montana*	*Cyclopogon congestus*
BM1	36.91 cC	94.44 aA	70.95 aB
½ [MS]	49.80 cB	77.38 bA	66.55 aA
½ [MS]–NO_3_	93.92 aA	64.30 bB	60.83 aB
Malmgren	76.89 bA	65.03 bA	34.17 bB

Different letters (lowercase in the column and uppercase in the lines) indicate significant differences by Tukey’s Test (*p* < 0.05).

## Data Availability

Data can be made available on request.
